# Sugar

**Published:** 1874-08

**Authors:** 


### SUGAR.

SWEETS are just as healthy in their place as roast beef. Pure candies promote digestion. It is all a mistake that they injure the teeth; it is not possible for them to do so. Men say that sugar rots the teeth. How do you know? Can you prove it? Bring your facts. It would be a singular thing if sweets were injurious to the health, because they are in everything we eat in the way of fruits, vegetables and the grains out of which we make our bread. Porterhouse steaks, and roast beef, and fried chickens have made more dyspeptics than sugar, by a thousand fold. Any injury resulting from the use of sugar, and candy, and preserves, is found in their being used too frequently, or in too large quantities; but everything we eat and drink is liable to the same objection. If taken before meals, or immediately after, both candies and nuts are promoters of digestion; the observation and the instincts of the civilized world on this point have led to the use of both at the end of meals; the reason was not known, but the fact was observed that both sweets and nuts came in comfortably after a good dinner; their very utility has made it fashionable.

It is the sugar in the food that keeps the infant warm; it would freeze, it would die without something sweet, hence nature has put sweetness in its food aliment, and given babies the world over an irrestrainable greediness for sweets. If sweets are taken only at meal time, not between, and not in excess, they will not only agree with any healthy stomach, but tend to make a man fat, as much as butter, for it is the carbon in each which the system uses. So abundant is sugar that it is found in almost all vegetable growths, even in the sawdust of our mills. Pour a little acid on lignite and sugar is an ultimate result, and very likely this is the reason of a report in England that a French house promises to furnish sugar at a penny a pound—which may be so, as sawdust costs nothing. There can be no doubt, in the beautiful language of the Episcopal prayer book, that the beneficent Creator has given us "all things richly to enjoy."

Everything that contains nutriment was made for our use, in moderation.

